# Rare Case of Metastatic Melanoma With Biventricular Cardiac Involvement: Diagnostic Challenges and Multimodal Management Strategies

**DOI:** 10.1016/j.jaccas.2025.105826

**Published:** 2025-11-26

**Authors:** Mohammed Najdat Seijari, Bana Sabbagh, Taraneh Zamani, Mohamad Hijazi, Godbless Ajenaghughrure, Fayaz Khan, Kamal Shemisa

**Affiliations:** a Department of Internal Medicine, Good Samaritan Hospital, Cincinnati, Ohio, USA; b Damascus University Hospital, Damascus, Syria; c Department of Cardiology, Good Samaritan Hospital, Cincinnati, Ohio, USA

**Keywords:** cardiac MRI (CMR), cardiac tumors, metastatic cardiac melanoma, multimodal imaging, PET-CT, surgical tumor debulking

## Abstract

**Background:**

Melanoma is an aggressive skin cancer with rising global incidence and potential for widespread metastasis, including to the heart. Cardiac involvement is rare, and standardized diagnostic and management strategies remain undefined.

**Case Summary:**

We present a patient with biventricular cardiac metastases from melanoma. Multimodal imaging—including transesophageal echocardiography, cardiac magnetic resonance imaging, and positron emission tomography–computed tomography—was essential in identifying cardiac tumor burden. The patient was treated with combination checkpoint inhibitor therapy (nivolumab and ipilimumab) and underwent surgical debulking. Clinical response was favorable.

**Discussion:**

This case underscores the importance of high clinical suspicion, especially as cardiac metastases may be clinically silent. It highlights the value of multimodal imaging in diagnosis, the role of systemic immunotherapy, and careful patient selection for surgery. Close monitoring during immunotherapy is vital given potential adverse effects.

**Take-Home Messages:**

Cardiac metastases from melanoma require a high index of suspicion and multimodal imaging for diagnosis. Multidisciplinary, individualized management is essential, especially when combining immunotherapy with surgical intervention.

## Graphical Abstract

**Figure:**
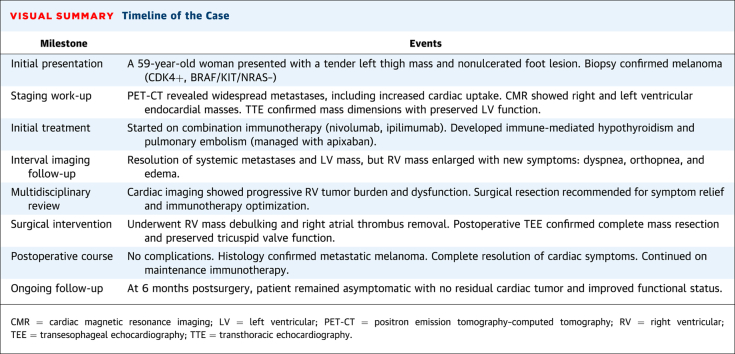

## History of Presentation

A 59-year-old woman presented with a 4-month history of a progressively enlarging, tender mass on the medial side of her left thigh. She also noted a mildly erythematous, nonulcerated lesion on the dorsal surface of her left foot. Physical examination revealed a palpable, firm, and tender mass in the left thigh without overlying skin changes, and a superficial lesion on the left foot. No other cutaneous abnormalities were observed.

**Take-Home Messages**

• In patients with metastatic melanoma, new or unexplained cardiac symptoms should prompt suspicion for cardiac involvement, which is frequently underdiagnosed.
• Early detection using multimodal imaging (TTE, CMR, and PET-CT), followed by timely management with immunotherapy and surgical debulking in selected cases, can significantly improve cardiac function, symptoms, and patient outcomes.

## Past Medical History

The patient had no prior history of melanoma or other malignancies. Her medical history was otherwise unremarkable.

## Differential Diagnosis

Initial differential diagnosis for the thigh and foot lesions included soft tissue sarcoma, cutaneous lymphoma, infectious granuloma, metastatic disease from an unknown primary, and primary cutaneous melanoma.

## Investigations

Both the thigh and foot lesions were excised, and histopathological analysis confirmed melanoma that was positive for CDK4 and negative for *BRAF*, *KIT*, and *NRAS* mutations.

**Figure 1:**
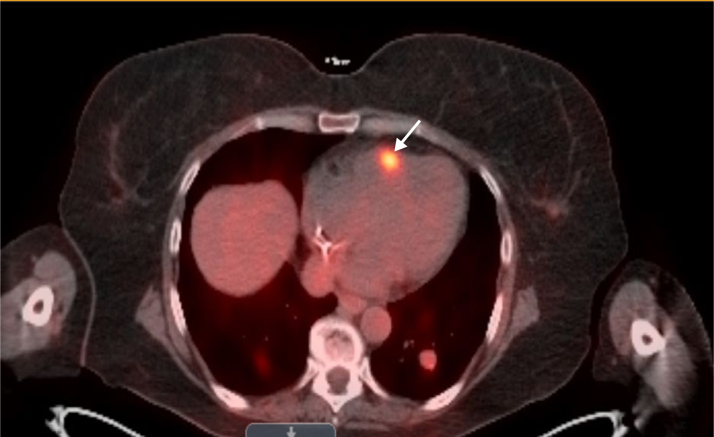
PET-CT Showing Multiple Nodular Cardiac Regions of Increased Metabolic Activity The arrow is pointing to the right ventricular mass. PET-CT = positron emission tomography–computed tomography.

**Figure 2:**
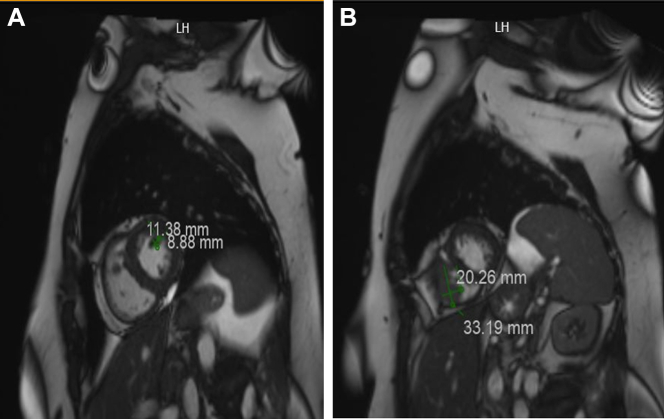
Findings on Cardiac Magnetic Resonance Imaging Cardiac magnetic resonance imaging showing (A) a 3.3-cm irregular mass in the right ventricle and (B) a 1.1-cm oval mass in the left ventricle.

**Figure 3:**
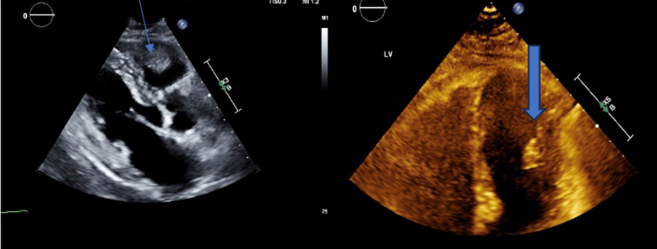
Findings on Transthoracic Echocardiography Cardiac echocardiography showing a 3.5-cm mass on the free wall of the right ventricle (thin blue arrow) and a 1.46-cm mass attached to the anterolateral papillary muscle in the left ventricle (thick blue arrow).

After diagnosis, positron emission tomography–computed tomography (PET-CT) (Figure 1) demonstrated widespread metastatic disease, including multiple hypermetabolic nodules and masses in the lungs, subcutaneous and intramuscular tissues of the chest and abdomen, right femoral condyle, and pelvic walls. Additionally, increased tracer uptake along the pericardium suggested possible cardiac involvement. Cardiac magnetic resonance imaging (CMR) (Figure 2) revealed a 3.3-cm irregular mass in the right ventricle (RV) and a 1.1-cm oval mass in the left ventricle, consistent with endocardial metastases. Subsequent two-dimensional transthoracic echocardiography (TTE) (Figure 3) confirmed normal left ventricular function and showed 2 fixed masses: a 3.5-cm mass on the free wall of the RV and a 1.46-cm mass attached to the anterolateral papillary muscle in the left ventricle.

**Figure 4:**
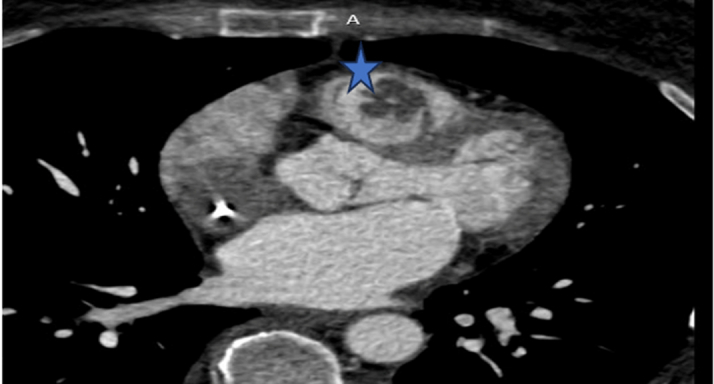
Computed Tomography Angiography Showing the Persistent Right Ventricular Mass (Star)

## Management

The patient commenced combination immunotherapy with nivolumab and ipilimumab, administered every 3 weeks for 4 cycles, followed by maintenance nivolumab every 2 weeks. The treatment was complicated by acquired hypothyroidism as a result of checkpoint inhibitor therapy. During this time, she developed a pulmonary embolism, which was managed with anticoagulation using apixaban.

After 18 months of immunotherapy, follow-up imaging indicated a significant treatment response, with a resolution of radiotracer uptake in the posterior right back, right inguinal lymph node, left thigh musculature, and left ventricular mass. However, she began to experience exertional shortness of breath, orthopnea, and lower limb edema.

The RV mass showed progression, with increased tracer uptake and size. Cardiac imaging demonstrated a large RV mass associated with RV dysfunction and impaired filling (Figure 4).

A multidisciplinary discussion concluded that surgical resection of the RV mass was necessary to relieve the newly developed symptoms, achieve debulking, enhance immunotherapy response, and obtain definitive histopathological confirmation of metastatic melanoma. Left heart catheterization showed no evidence of obstructive coronary artery disease and confirmed normal left ventricular function.

**Figure 5:**
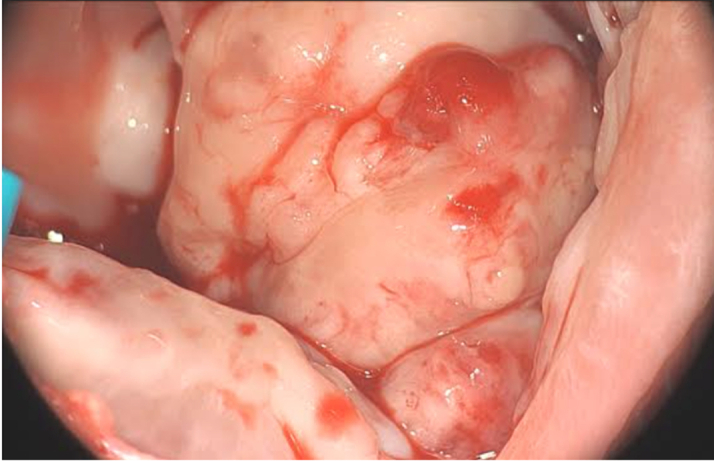
Right Ventricular Mass Resected During Surgery

**Figure 6:**
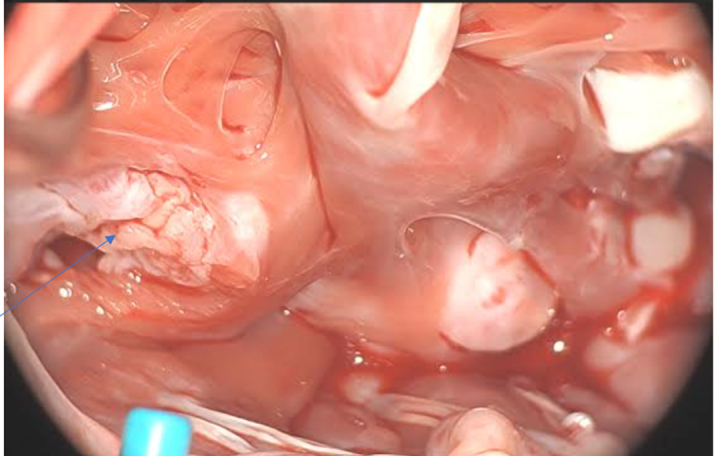
Several Small Implants Resected From the Right Ventricle (Thin Blue Arrow)

The patient underwent debulking surgery, during which 2 large RV tumor masses arising from the free wall and interventricular septum (Figures 5 and 6), along with several smaller implants, were resected. A right atrial thrombus was also removed. This procedure was completed without complications. Intraoperative inspection of the tricuspid valve revealed competency, and postoperative transesophageal echocardiography confirmed resolution of the RV mass and tricuspid valve integrity. Histology confirmed metastatic melanoma.

## Outcome and Follow-Up

The patient experienced no surgical complications, and 1-month postoperative transesophageal echocardiography showed no residual tumor. Her symptoms resolved completely after surgery, with improved exercise tolerance and quality of life. She continued immunotherapy for tumor control and was doing well at 6 months postprocedure.

## Discussion

Malignant melanoma is a highly aggressive skin cancer arising from melanocytes. Its etiology involves both environmental factors—primarily ultraviolet radiation—and genetic mutations. The disease is relatively common, more prevalent in older adults and males, and its incidence continues to rise. Globally, melanoma ranks as the 17th most common cancer per GLOBOCAN 2022 estimates.^1^

Although melanoma most often has cutaneous manifestation, it may also arise in mucosal sites (eg, head and neck), the uveal tract, and the gastrointestinal or genitourinary system. Its development reflects a complex interplay of host-related factors (eg, melanocytic nevi, family history, skin phototype, and genetic susceptibility) and environmental exposures, especially ultraviolet light, whether through sunlight, tanning beds, or PUVA (psoralen and ultraviolet-A radiation) therapy.

Hematogenous spread correlates with advanced disease and can involve multiple organs, including the lungs, brain, bones, and heart. Cardiac metastases are likely under-recognized; 1 study^2^ reported cardiac involvement in 50% of autopsies of patients with metastatic melanoma. In rare cases, cardiac metastasis has preceded the identification of a primary lesion. Melanoma is the second most common malignancy to metastasize to the heart.^3^

Clinical manifestations of cardiac involvement vary widely, from incidental findings on routine imaging to acute complications such as pericardial effusion (with or without tamponade), myocardial infarction, arrhythmias (including atrioventricular block, atrial fibrillation, or refractory ventricular tachycardia), and even sudden cardiac death.^3^^,^^4^ Thus, cardiac involvement should be suspected in melanoma patients with new cardiovascular symptoms. Conversely, cardiac masses should prompt a careful history and physical examination, including full skin inspection, to assess for melanoma.

Cardiac melanoma can affect all layers (endocardium, myocardium, and epicardium) and chambers, with the RV being most commonly involved (42%), followed by the left ventricle and right atrium (both 35%).^3^^,^^4^ In our patient, the tumor appeared confined to the endocardium.

Antemortem diagnosis of cardiac involvement is challenging. Imaging modalities such as TTE, CMR, and PET-CT play a vital role. TTE is helpful, especially for larger lesions, with a reported detection rate of 82%, although smaller masses may go unnoticed. CMR and PET-CT provide superior sensitivity and specificity, especially when used together. CMR can differentiate melanoma from other cardiac tumors owing to melanin's paramagnetic T1-shortening effect, reflected in lower T1 mapping values. PET-CT detection is enhanced by a high-fat, low-carbohydrate diet 24 hours prior and fasting overnight to reduce myocardial FDG (fluorodeoxyglucose) uptake, improving sensitivity in detecting smaller cardiac lesions.^5^ Thus, combining CMR with a TTE/PET-CT scan would be the best way to diagnose cardiac involvement with metastatic melanoma. Definitive diagnosis often requires histopathological confirmation via endovascular biopsy or surgical specimen analysis.^6^

Despite the important role of imaging for diagnosing cardiac involvement in suspected cases, no guidelines exist for screening asymptomatic patients. Routine cardiac imaging is generally unnecessary for asymptomatic melanoma cases, particularly in the lower stages (stage I and IIA). However, high-risk patients, such as those with widespread metastatic disease, may benefit from cardiac-specific imaging such as CMR, echocardiography, or PET-CT, although monitoring frequency remains undefined. For patients with diagnosed cardiac metastases, we recommend and are implementing a surveillance strategy of targeted echocardiography every 3 months and an annual PET scan.

The diagnosis and management of cardiac melanoma are both complex. Treatment options include immunotherapy, radiotherapy, and surgical resection. Current guidelines advocate for combination immunotherapy, specifically anti-CTLA4 and anti-PD1 agents such as ipilimumab combined with nivolumab, based on favorable outcomes in the CheckMate 067 and 069 trials, independent of *BRAF* mutation status. Nevertheless, it is crucial to closely monitor immune-related adverse events, including colitis, pneumonitis, myocarditis, and endocrinopathies (eg, hypothyroidism).^7^ Nassar et al^8^ examined the safety of immune checkpoint inhibitor therapy, particularly in patients with cardiac metastasis. Most reported adverse events were mild, primarily gastrointestinal related (eg, colitis and diarrhea), and included dermatitis and elevated liver enzymes. Cardiac-specific side effects necessitating cessation of immune checkpoint inhibitors occurred in only 2 cases. Overall, 32% of patients required steroid therapy because of side effects, while only 2 patients needed an additional immunosuppressant alongside steroids.^8^

Surgical resection is curative only in isolated cardiac metastasis, specifically in asymptomatic patients with clear margins. However, it can also enhance the quality of life or serve as a palliative approach to prevent imminent death from cardiac complications.^9^^,^^10^

Our patient initially underwent combination immunotherapy, resulting in the resolution of systemic metastases and the left ventricular mass. However, paradoxically, the RV mass enlarged, as imaging revealed an increased cardiac burden of metastasis, which led to symptomatic RV dysfunction. Subsequently, she underwent palliative surgical debulking, which led to complete resolution of her symptoms. She continued with immunotherapy and was doing well 6 months after the operation.

Overall, metastatic melanoma has a poor prognosis, and the presence of cardiac metastasis significantly worsens the 2-year survival rate.^4^ Early detection of cardiac melanoma metastases is critical and requires a high index of suspicion coupled with multimodal imaging. In carefully selected patients with limited tumor burden, surgical intervention can significantly improve quality of life and may offer a survival benefit.

## Conclusions

Melanoma frequently spreads hematogenously and can involve the heart, often without causing symptoms. Cardiac metastases should be considered in patients presenting with new cardiac complaints or unexplained cardiac masses. Early and accurate diagnosis relies heavily on multimodal imaging, particularly TTE, CMR, and PET-CT.

Systemic treatment continues to center around combination immunotherapy with ipilimumab and nivolumab, regardless of *BRAF* mutation status. Although surgical resection is rarely curative, it can provide symptom relief and improve outcomes in carefully selected patients with isolated or symptomatic cardiac involvement.

## Funding Support and Author Disclosures

The authors have reported that they have no relationships relevant to the contents of this paper to disclose.

**Visual Summary:** Timeline of the Case

Milestone	Events
Initial presentation	A 59-year-old woman presented with a tender left thigh mass and nonulcerated foot lesion. Biopsy confirmed melanoma (CDK4+, BRAF/KIT/NRAS–)
Staging work-up	PET-CT revealed widespread metastases, including increased cardiac uptake. CMR showed right and left ventricular endocardial masses. TTE confirmed mass dimensions with preserved LV function.
Initial treatment	Started on combination immunotherapy (nivolumab, ipilimumab). Developed immune-mediated hypothyroidism and pulmonary embolism (managed with apixaban).
Interval imaging follow-up	Resolution of systemic metastases and LV mass, but RV mass enlarged with new symptoms: dyspnea, orthopnea, and edema.
Multidisciplinary review	Cardiac imaging showed progressive RV tumor burden and dysfunction. Surgical resection recommended for symptom relief and immunotherapy optimization.
Surgical intervention	Underwent RV mass debulking and right atrial thrombus removal. Postoperative TEE confirmed complete mass resection and preserved tricuspid valve function.
Postoperative course	No complications. Histology confirmed metastatic melanoma. Complete resolution of cardiac symptoms. Continued on maintenance immunotherapy.
Ongoing follow-up	At 6 months postsurgery, patient remained asymptomatic with no residual cardiac tumor and improved functional status.

CMR = cardiac magnetic resonance imaging; LV = left ventricular; PET-CT = positron emission tomography–computed tomography; RV = right ventricular; TEE = transesophageal echocardiography; TTE = transthoracic echocardiography.
